# Echocardiographic left ventricular hypertrophy and geometry in Chinese chronic hemodialysis patients: the prevalence and determinants

**DOI:** 10.1186/s12872-022-02506-y

**Published:** 2022-02-16

**Authors:** Xinju Zhao, Li Zhu, Wenying Jin, Bing Yang, Yan Wang, Mengfan Ni, Yuchao Zhao, Liangying Gan, Li Zuo

**Affiliations:** 1 Department of Nephrology, Hemodialysis Center in Outpatient Building, Peking University People’s Hospital, 11 Xizhimennan Street, Xicheng District, Beijing, 100044 China; 2 Department of Cardiology, Peking University People’s Hospital, Beijing, China

**Keywords:** Maintenance hemodialysis, Left ventricular hypertrophy, Left ventricular mass index, Geometry, Risk factors

## Abstract

**Background:**

To investigate the prevalence of left ventricular hypertrophy (LVH) and explore left ventricular geometry in maintenance hemodialysis (MHD) patients, and to explore the risk factors of LVH which is an important predictor of cardiovascular events.

**Methods:**

The subjects were patients who are on MHD for more than 3 months in Peking University People's Hospital from March 2015 to February 2017. Demographic and clinical data were retrospectively collected. Left ventricular mass was measured by echocardiography. LVH is defined by Left ventricular mass index (LVMI) > 115 g/m^2^ for men and > 95 g/m^2^ in women. LVMI and relative wall thickness were used to determine left ventricular geometry. Logistic regression was used to analyze the risk factors of LVH.

**Results:**

Altogether, 131 patients including 77 males were enrolled. The median age was 60 (47, 69) years, with a median dialysis vintage of 48 (18, 104) months. There were 80 patients with LVH, the prevalence rate was 61.1%, and 66.3% of them were moderate to severe LVH. We found that (1) most of the patients were concentric hypertrophy; (2) one-third of the patients were concentric remodeling; (3) only 4 cases with normal geometry. The pre-dialysis serum sodium level and time average pre-dialysis systolic blood pressure (SBP) were independent risk factors of LVH.

**Conclusion:**

LVH is prevalent in MHD patients. Concentric hypertrophy and concentric remodeling are the most common geometric patterns. Attention should be paid to long-term pre-dialysis SBP management and pre-dialysis sodium control as they might be potentially modifiable risk factors for LVH.

## Background

Cardiovascular disease (CVD) is the main complication and primary cause of death in patients with end stage kidney disease (ESKD), accounting for about 50% of the total death [1, 2]. Left ventricular hypertrophy (LVH) plays a key role in the chain of cardiovascular events, which is closely related to the increase of cardiovascular events such as arrhythmia, atherosclerosis, stroke, and heart failure [3–5].

LVH is the most common cardiovascular abnormality in patients with chronic kidney disease (CKD) [6]. In non-dialysis dependent CKD patients, the prevalence of LVH is around 47%; while in ESKD patients, the prevalence rate of LVH can be as high as 75–89%. It is also reported that the risk of LVH increases with dialysis vintage and patients with LVH tend to progress.

LVH is often associated with a high risk of sudden cardiac death and is a predictor of cardiovascular death. The occurrence and continuous progression of LVH are associated with adverse cardiovascular prognosis and survival prognosis. However, the etiology of LVH in dialysis patients has not been fully clarified. The possible causes include anemia, hyperparathyroidism, toxin accumulation, malnutrition, etc., and volume overload and hypertension are still considered as important causes of LVH progression.

Diagnostic criteria for LVH had been updated. The prevalence of LVH in maintenance hemodialysis (MHD) patients under the relatively new diagnostic criteria is of great interest and value. The related research, especially for left ventricular geometry is few for Chinese MHD patients. Moreover, the risk factors deserve to be studied.

The purpose of this study was to explore the prevalence of LVH in Chinese MHD patients and the left ventricular geometry distribution determined by echocardiography, and to explore the risk factors of LVH to guide clinical treatment, so as to reduce the prevalence of LVH, and reduce the risk of cardiovascular events and cardiovascular death.

## Methods

### Study design and participants

This study was a single center, retrospective cohort study. Eligible MHD patients in Peking University People's Hospital from March 2015 to February 2017 were enrolled. The inclusion criteria were: (1) who received MHD for more than 3 months; (2) who had echocardiography record in this period. The exclusion criteria were: (1) who had any active infections (bacterial and viral infections); (2) who had myocardial infarction, acute heart failure or stroke event occurred within 1 months of the echocardiography test; (3) History of malignant tumors, except for the following cases: tumors identified as cured or relieved for more than 5 years, cutaneous basal cells or squamous cell carcinoma or carcinoma in situ has been radically resected.

This study was conducted in accordance with the Declaration of Helsinki (as revised in 2013). The study was approved by the Ethics Committee of Peking University People’s Hospital (ethical approval number: 2019PHB203-01). As this study was a retrospective observational cohort study without any intervention, informed consent was exempted by the Ethics Committee.

### Demographic and clinical data collection

Demographic characteristics including age, gender, body mass index (BMI), dialysis vintage, assigned primary ESKD causes, dry weight, laboratory values including hemoglobulin, pre-dialysis biochemistry (serum creatinine, blood nitrogen, urine acid, calcium, phosphorus, potassium, sodium, chloride, CO_2_ binding capacity, glucose, total protein, albumin, total cholesterol, triglyceride, parathyroid hormone), Kt/v, nitrogen reduction rate (URR), and clinical data, such as average intradialytic weight loss (IDWL) of 3 consecutive sessions, monthly average of pre-dialysis and post-dialysis systolic and diastolic blood pressure (SBP and DBP) were retrospectively collected.

### Echocardiographic examination

Transthoracic echocardiographic examinations were performed for all subjects (GE Vivid 7 or Vivid E9, GE Vingmed, Horten, Norway; ALOKA Prosound F75, Tokyo, Japan) with a 3–6 MHz phased array transducer. Standard two-dimensional echocardiography with Doppler examination was performed and measurements were obtained according to the guidelines of American Society of Echocardiography [7]. Left ventricular mass was measured by echocardiography and left ventricular mass index (LVMI) was calculated [8]. LVH is defined by LVMI > 115 g/m^2^ for men and > 95 g/m^2^ in women [9]. According to LVMI, LVH was further divided into mild, moderate, and severe category (Table 1) [9]. LVMI and relative wall thickness (RWT) were used to group the left ventricular geometry [9]. RWT is calculated by the formula (2 × posterior wall thickness)/(LV internal diameter at end-diastole) [10] and permits categorization of an increase in LV mass as either concentric (RWT > 0.42) or eccentric (RWT ≤ 0.42) hypertrophy and allows the identification of concentric remodeling (normal LV mass with increased RWT, Table 2) [9].

**Table 1:** The LVH severity category

	Female	Male
	LVMI (g/m^2^)	LVMI (g/m^2^)
Normal	43–95	49–115
Mild	96–108	116–13,127
Moderate	109–121	132–148
Severe	≥ 122	≥ 149

**Table 2:** Left ventricular geometry classification

LV geometry	LVMI (g/m^2^)	RWT
Normal	M ≤ 115; F ≤ 95	≤ 0.42
Concentric remodeling	M ≤ 115; F ≤ 95	> 0.42
Concentric hypertrophy	M > 115; F > 95	> 0.42
Eccentric hypertrophy	M > 115; F > 95	≤ 0.42

*M* Male, *F* Female

### Statistical analysis

The continuous variables of normal distribution were expressed as mean ± standard deviation, and the comparison of mean between groups was conducted by independent sample t test. The continuous variables of non-normal distribution were expressed by median (25th, 75th). Wilcoxon rank sum test was used for comparisons. Categorical variables were expressed as rates or percentages, and comparisons between groups were performed by chi square test. Stepwise multivariate unconditional logistic regression analysis (*P* < 0.05) was conducted to determine the independent risk factors of LVH from all clinically relevant variables. We stratified continuous variables considered as risk factors into categorical variables to investigate the differences in LVH risk among subgroups of each variable. The patients were stratified into two groups according to the mean value of serum sodium level before dialysis in our study (< 138 mmol/L and ≥ 138 mmol/L). The patients also stratified according to their average pre-dialysis systolic blood pressure. *P* < 0.05 was statistically significant. The statistics were completed by SAS software (version 9.4, SAS Institute, Cary, NC, USA).

## Results

**Fig. 1:**
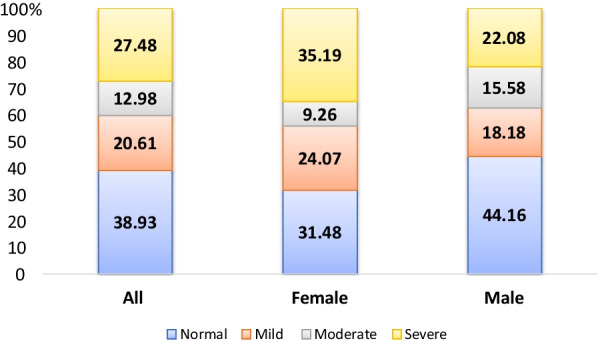
The distribution (%) of left ventricular hypertrophy severity in MHD patients

A total of 131 patients with 77 males and 54 females were enrolled. The median age was 60 (47, 69) years old, with a median dialysis vintage of 48 (18, 104) months (Table 3). There were 80 patients with LVH. The prevalence of LVH in this single center was 61.1%. Among LVH patients, 66.3% of them were moderate or severe LVH (Fig. 1). Compared with patients without LVH, patients with LVH had lower level of total protein (71.90 ± 5.32 vs. 69.90 ± 4.05, *P* = 0.019), higher potassium level (4.71 ± 0.73 vs. 5.05 ± 0.74, *P* = 0.014), higher Pre-dialysis SBP and Post-dialysis SBP. The median dialysis vintage was 56 months in the LVH group and 40 months in the non-LVH group, but not statistically significant (*P* = 0.196).

**Table 3:** Baseline characteristics of MHD patients with and without LVH

Variables	All (n = 131)	LVH (n = 80)	Non-LVH (n = 51)	*P* value
*Demographics*				
Age (years)	60 (47, 69)	59.5 (49.5,70.5)	61 (46, 68)	0.479
Males (%)		53.75	66.67	0.151
Vintage (months)	48 (18, 104)	56(24, 114)	40 (17, 98)	0.196
BMI(kg/m^2^)	22.6 (20.3, 25.3)	22.8 (20.1, 25.4)	22.1 (20.5, 24.9)	0.489
*ESRD causes (%)*				–
Glomerulonephritis	49.6	53.8	43.1	
Diabetic nephropathy	16.8	15.0	19.6	
Hypertensive nephropathy	11.5	6.3	19.6	
Others	22.1	25.0	17.7	
*Laboratory tests*				
Hgb (g/l)	114(108, 121)	115.5(108, 121)	113(107, 120)	0.283
Alb (g/l)	39.7(37.8, 41.8)	39.2 ± 3.2	39.7 ± 3.8	0.112
BUN (mmol/l)	28.2(23.5, 32.4)	28.8(23.5, 33.5)	27.6(22.9, 29.9)	0.132
Creatine (μmol/l)	1004 (852, 1191)	1011 (880, 1191)	983 (837, 1188)	0.513
UA (μmol/l)	449.6 ± 94.5	450 ± 101.8	448 ± 82.6	0.884
adjusted Ca (mmol/l)	9.4 ± 0.7	9.4 ± 0.7	9.4 ± 0.7	0.716
P (mmol/l)	1.62 ± 0.54	1.62 ± 0.56	1.64 ± 0.51	0.817
PTH (μg/ml)	173.0(96.4, 337.6)	186.5(107.4, 361.7)	163.7(58.1, 300.7)	0.186
LDL (mmol/l)	2.28 ± 0.81	2.21 ± 0.83	2.37 ± 0.76	0.271
HDL (mmol/l)	0.98(0.82, 1.25)	0.98(0.79, 1.25)	1.00(0.84, 1.24)	0.500
Total cholesterol (mmol/l)	4.11(3.51, 4.81)	4.27(3.72, 4.84)	4.00(3.46, 4.74)	0.402
Triglyceride (mmol/l)	1.94 (1.29, 2.75)	1.94 (1.22, 2.62)	1.90 (1.31, 3.16)	0.500
Total Protein (g/l)	70.68 ± 4.67	69.9 ± 4.05	71.9 ± 5.32	0.019
Glucose (mmol/l)	6.71(5.41, 9.18)	6.77(5.72, 9.02)	6.65(5.30, 9.68)	0.992
Potassium (mmol/l)	4.92 ± 0.75	5.05 ± 0.74	4.71 ± 0.73	0.014
Na (mmol/l)	138.0 ± 3.4	138.2 ± 3.3	137.6 ± 3.6	0.354
CO_2_CP (mmol/l)	23.8 ± 3.0	23.5 ± 2.9	24.4 ± 3.2	0.109
*Dialysis related indices*				
spKt/V	1.49 ± 0.26	1.47 ± 0.27	1.51 ± 0.24	0.378
URR (%)	70.46 ± 7.26	70.03 ± 7.04	71.13 ± 7.63	0.411
Fistula use (%)	84.7	85.0	84.3	1.000
Intradialytic weight loss (kg)	2.7 (2.3, 3.2)	2.7 (2.3, 3.3)	2.5 (2.0, 3.0)	0.145
*Clinical and echocardiography indices (%)*				
Pre-dialysis SBP (mmHg)	151 ± 16	154 ± 14	146 ± 17	0.004
Pre-dialysis DBP (mmHg)	76 ± 11	77 ± 12	75 ± 10	0.367
Post-dialysis SBP (mmHg)	145 ± 16	148 ± 15	141 ± 18	0.008
Post-dialysis DBP (mmHg)	79 ± 11	80 ± 12	79 ± 11	0.702
LVM (g)	197.5 (161.7, 262.0)	243.5 (199.1, 280.8)	149.0 (123.6, 181.9)	< 0.001
LVMI (g/m^2^)	115.0 (95.9, 144.7)	133.1 (120.9, 161.1)	87.7 (77.2, 97.7)	< 0.001
LVEF (%)	65.3 (59.3, 71.1)	63.3 (54.8, 69.2)	68.6 (63.1, 72.0)	0.003
MPA (cm)	2.3 (2.1, 2.5)	2.3 (2.2, 2.7)	2.2 (2.1, 2.4)	0.013
LAD (cm)	3.8 (3.4, 4.1)	3.9 (3.5, 4.4)	3.9 (3.3, 4.0)	0.005
IVSd (cm)	1.0 (0.9, 1.1)	1.1 (1.0, 1.2)	0.9 (0.8, 1.0)	< 0.001
LVIDd (cm)	5.0 (4.6, 5.4)	5.1 (4.9, 5.7)	4.5 (4.3, 5.0)	< 0.001
LVIDs (cm)	3.2 (2.8, 3.6)	3.4 (3.1, 3.9)	2.9 (2.7, 3.2)	< 0.001
PWT (cm)	0.97 (0.88, 1.10)	1.00 (0.90, 1.10)	0.90 (0.80, 1.00)	< 0.001

*BMI* body mass index, *Hgb* haemoglobin, *Alb* albumin, *spKt/V* single-pooled Kt/V, *stdKt/V* standardized Kt/V, *LVM* left ventricular mass, *LVMI* left ventricular mass index, *LVEF* left ventricular ejection fraction, *MPA* main pulmonary artery diameter, *LAD* anteroposterior diameter of left atrium, *IVSd* interventricular septum at end-diastole, *LVIDd* left ventricular internal diameter at end-diastole, *LVIDs* left ventricular internal diameter at end-systole, *PWT* inferolateral wall thickness

**Fig. 2:**
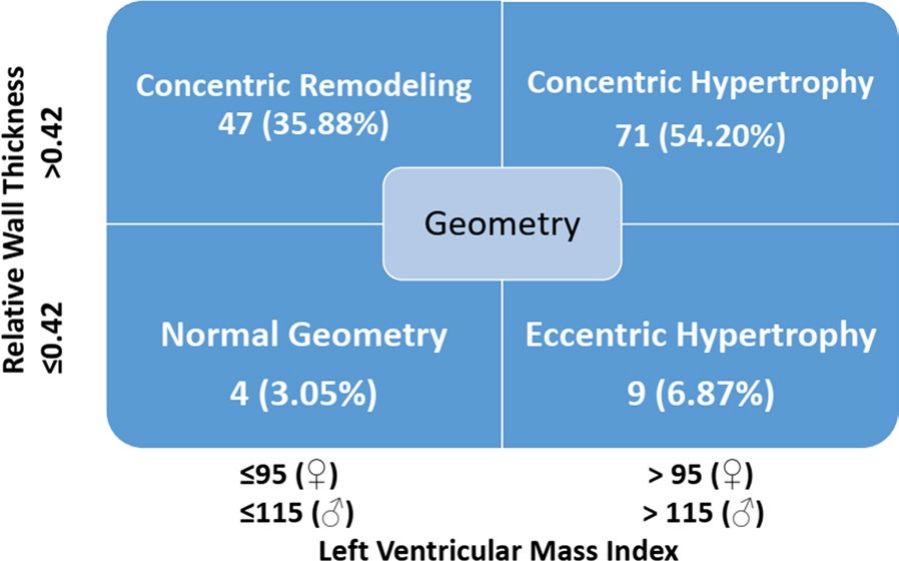
The classification of Left ventricular geometry for MHD patients

The left ventricular geometry showed that there were 47 cases with concentric remodeling, 71 cases with concentric hypertrophy, 9 cases with eccentric hypertrophy, and only 4 cases with normal left ventricular geometry (Fig. 2).

For echocardiography indies, compared with patients in non LVH group, LVEF was lower, while main pulmonary artery diameter, anteroposterior diameter of left atrium, interventricular septum at end-diastole, left ventricular internal diameter at end-diastole, left ventricular internal diameter at end-systole, and inferolateral wall thickness were greater in LVH group.

Stepwise logistic regression analysis showed that pre-dialysis serum sodium level and pre-dialysis systolic blood pressure were independent risk factors of LVH, and OR values were 1.136 (95% CI 1.005–1.284) and 1.047 (95% CI 1.017–1.079), respectively. Further stratified analysis of pre-dialysis serum sodium and blood pressure showed that patients with Na ≥ 138 mmol/L or systolic blood pressure higher than 150 mmHg had an increased risk of LVH (Table 4).

**Table 4:** Stepwise multivariate logistic regression analysis and stratified analysis of risk factors

	OR value	95%CI	*P* value
Pre-dialysis sodium(mmol/L)	1.136	1.005–1.284	0.041
< 138	ref	ref	ref
≥ 138	1.146	1.012–1.296	0.031
Pre-dialysis SBP (mmHg)	1.047	1.017–1.079	0.002
≤ 130	ref	ref	ref
131–139	1.949	0.300–12.651	0.117
140–149	7.670	1.500–39.210	0.243
150–159	12.891	2.611–63.638	0.010
≥ 160	12.412	2.461–62.605	0.018

## Discussion

In the current study, we found that the prevalence of LVH is high in MHD patients, and most of them are concentric hypertrophy, which is consistent with previous studies. Patients with complete normal cardiac geometry is rare. Even in MHD patients without LVH, concentric remodeling is also common. However, the prevalence of eccentric hypertrophy seems to be a little lower than reported, which might be influenced by the different study population.

In literature, LVH is present in 68–89% of incident HD patients [11–14]. Chronic volume overload, hypertension, high output of AV fistulae, anemia, and uremic toxin accumulation all contribute to LVH in MHD [15]. Excessive sodium can induce water retention and hence excessive volume. Volume overload has been recognized as one of the main etiologies of hypertension in HD patients [16]. Reversely, accurate assessment and management of volume status was shown to be associated with reduced volume overload and LVH improvement [17]. The correlation between dialysis vintage and LVH is supported by evidence [18]. However, the difference for dialysis vintage between the LVH group and non-LVH group did not reach the statistical significance at *P* < 0.05 in our study, which might be limited by the relatively small sample size.

Although regression can be observed in a minority of patients, more often, LVH progresses. Zoccali et al. followed up 161 MHD patients without chronic congestive heart failure (LVEF > 35%) for 18 months. The researchers found that LVMI increased by 6.7% at the end of follow-up [19]. Levin et al. conducted a prospective multicenter cohort study in Canadian patients with a total of 246 patients included. In their study, one quarter of patients were found to have increased LVH after 1 year of follow-up [20]. Foley et al. followed up 596 newly dialyzed patients (dialysis vintage < 18 months) without obvious heart disease and cardiac enlargement. Their study found that no matter what treatment was randomly given, the average LVMI of patients showed a trend of progression. LVMI was 114 g/m^2^ at baseline, 121 g/m^2^ at week 24, 123 g/m^2^ at week 48, and 128 g/m^2^ at week 96 (*P* < 0.001) [21].

LVH can be served as a risk factor for cardiac and all-cause mortality [22]. Stack et al. found that in the incident dialysis patients, the survival rate of patients with LVH at 6 months, 12 months and 24 months was worse than that of patients without LVH, and the relative risk of death was 1.61, 1.36 and 1.29 respectively [23]. LVH also significantly increases the risk of heart failure [24]. The risk of heart failure in patients with cardiotropic LVH and left ventricular dilatation was 3.7 times and 4.7 times higher than those with normal measurement [25]. Researchers found that 10% decrease of LVM was independently associated with 22% and 28% decreased risk of all-cause and cardiovascular mortality [26]. The above publications reveal that LVH is associated with worse clinical outcomes and might be a modifiable risk factor for mortality.

How to delay or reverse LVH is the key and difficult point in the treatment of maintenance hemodialysis patients. However, in addition to intensive dialysis, treatment interventions are very limited [3]. It has been a long time since scholars recognized hypertension as a risk factor for LVH in hypertensive and CKD populations [27–30]. However, for MHD patients to what extent the blood pressure is associated with LVH and which blood pressure level is associated an increased risk is not well studied [31]. Our findings suggested that the time average pre-dialysis SBP was independently associated with LVH. As far as we know, this is the first manuscript introducing time average blood pressure concept. LVH is a time dependent cumulative effect of volume and post cardiac overload. Not surprisingly, some research with random blood pressure levels showed a negative result. Moreover, our stratified analysis showed that only the time average pre-dialysis SBP > 150 mmHg was associated with an increased risk of LVH. This result suggested that the target of pre-dialysis SBP for MHD patients should not exceed 150 mmHg in terms of reign LVH. Nowadays, some research and guidelines recommend to measure blood pressure on inter-dialytic days, based on the evidences that inter-dialytic blood pressure measurements were shown to be better associated with 44-h ambulatory blood pressure and mortality in HD patients. Whether the inter-dialytic blood pressure will be a risk factor for LVH and surpass pre-dialysis blood pressure is warrant further studies.

In our study, we also found that the pre-dialysis serum sodium level is an independent risk factor of LVH. A large amount of empirical evidence has linked sodium excess to increased incidence and mortality of cardiovascular disease in HD patients. Sodium retention is common in patients undergoing dialysis. LVH could be mediated by sodium retention through hypertension, increased intravascular volume, increased afterload and cardiac traction. We found the risk of LVH was increased in patients with serum sodium ≥ 138 mmol/L in our study, which is our median value of sodium level and also a well-used cut off value to assess prognosis [32–34]. Our finding is consistent with former study [34]. Moreover, scholars suggest that adjusting dialysate sodium concentration might be a simple method to ameliorate sodium retention for HD patients. It is expected to improve sodium regulation in HD patients by lowering dialysate sodium, so as to reduce chronic fluid and sodium overload. The value 138 mmol/L is the routine prescription sodium concentration in dialysate in our hemodialysis center. For dialysis patients, the optimal serum and dialysate sodium level is always on a debate focus [35]. Our finding suggests that the serum sodium level should not exceed 138 mmol/L in MHD patients.

In other studies, researchers found that intradialytic weight loss or inter-dialytic weight gain was associated with LVH [36]. However, in our stepwise regression analysis, we were not able to identify it as a risk factor. We use the average value of 3 consecutive HD sessions which may not be so representative of a long-time volume load. Whether monthly, quarterly or even yearly average IDWL will be a risk factor of LVH is worthy further research. Some studies suggest that cardiac valvular calcification may be one of the risk factors for left ventricular hypertrophy, while our results have no statistical difference [37]. It is worthy further study.

However, our study has several limitations. Firstly, this is a single center, retrospective, observational study which might has inherent shortcomings such as selection bias and confounding factors. Secondly, the small sample size in our study makes it impossible for us to perform more detailed subgroup analysis. To solve this problem, we have used a relatively more flexible inclusion criteria of enrolling patients being on HD treatment for more than 3 months instead of 6 months or longer. This criterion enabled us to include as many eligible participates as possible, and guaranteed that they are all MHD patients as well. Usually, being on HD treatment more than 3 months is recognized as MHD patients, and most studies in HD population enrolled participants on HD more than 3 months [38–40]. Furthermore, some research suggested that 3 months might be long enough to make a difference on the progression of LVH in HD population [41]. Therefore, we believe that this design will not affect our main findings though further large scale and well-designed prospective study is needed. Thirdly, comorbidity and medicine information were not collected in this study which might cause bias. Even though, the results of this study have value for us to understand the LVH epidemiology and to explore potential modifiable risk factors of LVH, and hence might be able to reduce the LVH risk and improve patients’ survival.

## Conclusion

In conclusion, the prevalence of LVH in MHD patients in China is high, and normal geometry is rare. Routine echocardiography examination to detect LVH and left ventricular geometry in MHD patients is highly recommended. We revealed that pre-dialysis SBP and pre-dialysis sodium might be potential modifiable risk factors for LVH. We suggest that the pre-dialysis SBP should not exceed 150 mmHg and pre-dialysis serum sodium should be less than 138 mmol/L in MHD patients for cardiac protection, which warrants further study.

## Data Availability

The datasets used and/or analyzed during the current study are available from the corresponding author on reasonable request.

## Abbreviations

BMI: Body mass index
CKD: Chronic kidney disease
CVD: Cardiovascular disease
DBP: Diastolic blood pressure
ESKD: End stage kidney disease
IDWL: Intradialytic weight loss
LVH: Left ventricular hypertrophy
LVMI: Left ventricular mass index
MHD: Maintenance hemodialysis
RWT: Relative wall thickness
SBP: Systolic blood pressure
URR: Nitrogen reduction rate
